# Silk fibroin nanofibers: a promising ink additive for extrusion three-dimensional bioprinting

**DOI:** 10.1016/j.mtbio.2020.100078

**Published:** 2020-09-19

**Authors:** S. Sakai, A. Yoshii, S. Sakurai, K. Horii, O. Nagasuna

**Affiliations:** a Division of Chemical Engineering, Department of Materials Engineering Science, Graduate School of Engineering Science, Osaka University, 1-3 Machikaneyama-Cho, Toyonaka, Osaka, 560-8531, Japan; b Nagasuna Mayu Inc., Kyotango, Kyoto, 629-3101, Japan

**Keywords:** 3D printing, Tissue engineering, 3D printer, Additive manufacturing, Bombyx mori silk fiber

## Abstract

Here, we investigated the usefulness of silk fibroin nanofibers obtained via mechanical grinding of degummed silkworm silk fibers as an additive in bioinks for extrusion three-dimensional (3D) bioprinting of cell-laden constructs. The nanofibers could be sterilized by autoclaving, and addition of the nanofibers improved the shear thinning of polymeric aqueous solutions, independent of electric charge and the content of cross-linkable moieties in the polymers. The addition of nanofibers to bioinks resulted in the fabrication of hydrogel constructs with higher fidelity to blueprints. Mammalian cells in the constructs showed >85% viability independent of the presence of nanofibers. The nanofibers did not affect the morphologies of enclosed cells. These results demonstrate the great potential of silk fibroin nanofibers obtained via mechanical grinding of degummed silkworm silk fibers as an additive in bioinks for extrusion 3D bioprinting.

## Highlights

• Silk fibroin nanofibers are sterilizable by autoclaving.
• Shear thinning of polymer solutions was enhanced by silk fibroin nanofibers.
• Inks containing silk fibroin nanofibers realize bioprinting with high printability.
• Silk fibroin nanofibers in bioinks are cytocompatible.
• Silk fibroin nanofibers are inert toward cells in bioprinted hydrogels.

## Graphical abstract

**Figure:**
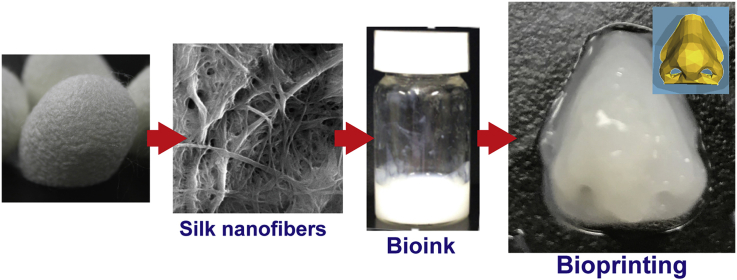

## 1 Introduction

Three-dimensional (3D) bioprinting is the technique of 3D printing of cell-laden constructs based on preprogrammed digital blueprints. Recently, 3D bioprinting has attracted increasing attention as an unparalleled manufacturing technology that enables the creation of cell-laden 3D constructs mimicking functional tissues and organs with complex structures [[1], [2], [3], [4]]. While bioprinting is still in an early stage of development, cell-laden constructs obtained using this technology are expected to be important for use as *in vitro* models for drug screening and as tissues for transplantation and regenerative medicine [[1], [2], [3], [4]]. Bioprinting is broadly classified into two approaches, based on whether or not supporting hydrogels are used [5,6]. In the latter approach, individual cells or pellets of cells are assembled using, for example, magnetic force and microneedles [[7], [8], [9]]. In the approach of the use of supporting hydrogels, cells are printed in a solution called the ‘bioink’ which can be cross-linked or stabilized during or immediately after printing [1,2]. An advantage of the approach is the ease of creating 3D constructs with preferred shapes and forms [6]. In addition, cellular behaviors such as proliferation, elongation, and differentiation can be controlled by using appropriate hydrogels as well as the mechanical properties of 3D constructs [4].

Existing bioprinting systems for the approach of the use of supporting hydrogels can primarily be classified into three types: inkjet, extrusion, and laser-assisted bioprinting [4]. Among them, extrusion printing, in which the bioink is extruded layer-by-layer from a micronozzle of a printer, is the most widely investigated type [4]. In this system, the flow characteristics of the bioink determine the printability [1,2]. In general, the printability can be improved by increasing the viscosity because this enables the extruded bioink to better hold its shape [1,2]. However, an increase of viscosity increases the shear stress during printing, which can damage cells contained in the bioink, and the resistance to flow, which can increase the risk of the micronozzle clogging [1,2]. A promising approach for improving printability without reducing cell viability is the use of nanomaterials as additives; these induce a decrease of viscosity with increasing applied force (shear thinning) [1,2,10,11]. Various nanomaterials, such as nanosilicate [12] and nanohydroxyapatite [13], have been used for this purpose. Cellulose nanofibers also have been widely investigated for this purpose. The addition of cellulose nanofibers, including oxidized cellulose nanofibers, has been shown to improve printability by the enhancing shear thinning behavior, and to increase the compressive modulus of the resultant hydrogels [[14], [15], [16], [17]]. However, the development of novel additives for bioinks that are biocompatible and result in the shear thinning behavior is still an important and challenging issue for the progress of bioprinting. This is because the required characteristics of bioinks are different in each application. Besides, materials that can be obtained via simple processes are more attractive.

**Fig. 1:**
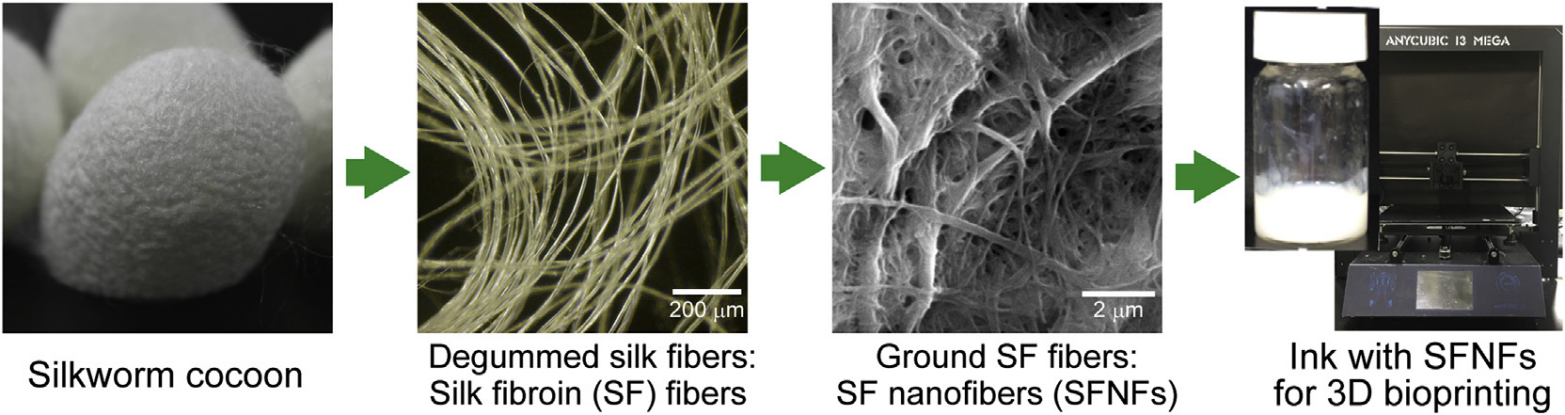
Images of silkworm cocoon, degummed silk fibroin fibers, silk fibroin nanofibers (SFNFs) obtained by grinding of silk fibroin fibers dispersed in water, and ink-containing SFNFs used for 3D bioprinting. 3D, 3-dimenssional.

Here, we show the great potential of silk fibroin nanofibers (SFNFs) obtained via mechanical grinding of degummed silkworm silk fibers as an additive for bioinks containing cells for extrusion-based bioprinting (Fig. 1). Zhang et al. [18] reported a 3D printing of acellular chitosan hydrogel constructs using an acetic acid solution containing chitosan and silk nanofibers. Because of its acid nature, the solution can not be applied to bioinks containing cells. As far as the authors are aware, no previous reports have revealed the usefulness of SFNFs in the fabrication of cell-laden constructs by 3D bioprinting. In recent years, the use of silkworm silk, composed of two major proteins—silk fibroin and sericin—has been discussed extensively in a wide range of fields because of its excellent biocompatibility, robust mechanical performance, ease of processing, and sufficient supply from the mature sericulture industry [19]. In medicine, silk is widely used in sutures, surgical meshes, and fabrics, and clinical trials are being conducted for its use in wound healing and tissue engineering [20]. For clinical use, removal of sericin from silk by degumming is essential because sericin has traditionally been linked to the inflammatory response reported for virgin silk [20,21]. SFNFs have often been prepared based on the electrospinning of silk fibroin solutions obtained by dissolution through the decomposition of the β-sheet structure of silk fibroin that contributes to the mechanical robustness of native silk fibers [[22], [23], [24]]. However, obtaining SFNFs by mechanical disintegration of degummed silk fibers is simpler and less energy- and time-consuming than preparation of SFNFs by electrospinning [25].

Recently, the fabrication of cell-laden hydrogel constructs through enzymatic cross-linking using a variety of fabrication processes, including 3D bioprinting, has attracted attention in the tissue engineering field [[26], [27], [28], [29], [30], [31], [32]]. In this study, the effect of SFNFs on shear thinning of polymer solutions was investigated using solutions dissolving chitosan, alginate (Alg), poly(vinyl alcohol) (PVA), and derivatives of hyaluronic acid (HA) and gelatin. The derivatives of HA and gelatin possessed phenolic hydroxyl (Ph) moieties cross-linkable through horseradish peroxidase (HRP)–catalyzed reaction. Then, the effects of SFNFs on 3D bioprinting were evaluated using solutions containing the HA derivative alone or mixed with the gelatin derivative.

## 2 Materials and methods

### 2.1 Materials

SFNFs of 100–300 nm in diameter and 4–7 μm in length (Fig. 1) were obtained from Nagasuna Mayu (Kyoto Japan). The SFNFs were produced by grinding silk fibroin fibers dispersed in water using a grinder (MKCA 6–3, Masuko Sangyo Co., Saitama, Japan) based on a reported method [25]. Sodium HA (molecular weight [MW]: *circa* 1000 kDa) was purchased from Kewpie (Tokyo, Japan). PVA (MW: 14.6–18.6 kDa) and gelatin from the bovine skin (type B) were obtained from Sigma-Aldrich (St. Louis, MO). Sodium Alg (MW: 70 kDa, high content of guluronic acid) and chitosan (PSH; MW: 500–800 kDa, deacetylation >80%) were obtained from Kimica (Tokyo, Japan) and Yaizu Suisan Kagaku (Shizuoka, Japan), respectively. *N*-hydroxysulfosuccinimide (NHS), HRP, and H_2_O_2_ aqueous solution (31 w/w%) were purchased from Fujifilm Wako Pure Chemical Industries (Osaka, Japan). 1-ethyl-3-(3-dimethylaminopropyl)carbodiimide monohydrochloride (EDC) was obtained from the Peptide Institute (Osaka, Japan). Derivatives of HA and gelatin possessing Ph moieties (denoted HA-Ph and gelatin-Ph, respectively) were obtained by conjugating amino moieties of tyramine with carboxyl moieties of HA and amino moieties of gelatin with carboxyl moieties of 3-(4-hydroxyphenyl)propanoic acid using EDC and NHS, as previously reported [33,34]. The contents of Ph moieties in HA-Ph and gelatin-Ph were 1.43 × 10^−4^ mol-Ph/g and 2.25 × 10^−4^ mol-Ph/g, respectively. Mouse fibroblast cell line 10T1/2 cells were obtained from the RIKEN Cell Bank (Ibaraki, Japan) and grown in the Dulbecco's modified Eagle's (DME) medium containing 10 vol% fetal bovine serum in humidified air at 37 °C in a 5% CO_2_ incubator.

### 2.2 Viscoelastic property measurement

The viscoelastic properties of solutions were measured using a rheometer (HAAKE MARS III, Thermo Fisher Scientific, MA) equipped with a parallel plate of 50 mm diameter with a 0.5-mm gap. HA, PVA, Alg, chitosan, HA-Ph, and gelatin-Ph were used as substrate(s) of the solutions. The content of SFNFs in solutions was varied between 0 w/v% and 1.0 w/v%. Phosphate-buffered saline (PBS) (pH 7.4) was used as the solvent for the solutions except when chitosan was a substrate; chitosan was dissolved in aqueous HCl solution (pH 4.6).

### 2.3 Gelation time measurement

Gelation times of 1.5 w/v% HA-Ph solutions containing 0–1.0 w/v% SFNFs and 5 U/mL HRP were measured based on a reported method [35]. Briefly, each solution (0.4 mL) was poured into a well of a 24-well plate and stirred using a magnetic stirrer bar. Then, air containing 14 ppm H_2_O_2_ obtained by bubbling air in 1 M H_2_O_2_ aqueous solution flowed onto the solution surface. Gelation was indicated by the hindrance of magnetic stirring and swelling of the solution surface.

### 2.4 Mechanical property measurement

Mechanical properties of hydrogels were determined by measuring the repulsion forces toward compression (10 mm/min) of cylindrical hydrogels of the 15-mm diameter and 3-mm height using a Table-Top Material Tester (EZ-test, Shimadzu, Kyoto, Japan) equipped with a probe of 20-mm diameter. The hydrogels were obtained from solutions containing polymer(s), 0–1.0 w/v% SFNFs, and 5 U/mL HRP by exposure to air containing 14 ppm H_2_O_2_. Young's moduli of specimens were calculated from the data for 0.5%–3% strain.

### 2.5 Printability measurement

A computer-connected extrusion 3D printing system developed by modifying a commercial 3D printing system (Anycubic i3 Mega; Anycubic, Guangdong, China) was used for 3D printing. The printing system consisted of a syringe pump for flowing ink, a 27-gauge stainless steel needle for extruding the ink, a bubbling system for supplying air containing 14 ppm H_2_O_2_, and a stage for layering the extruded ink. The flow rates of inks in the needle and the moving speed of the stage were fixed at 22 mm/s. The printing of cell-laden 3D hydrogel constructs was performed in a biological safety cabinet. Inks containing 1.5 w/v% HA-Ph or 0.1 w/v% HA-Ph + 0.9 w/v% HA + 1.0 w/v% gelatin-Ph, and free of SFNFs or containing 1.0 w/v% SFNFs were used. The inks contained HRP (5 U/mL).

### 2.6 Cell studies

Cell-laden disk-shaped hydrogel constructs (8 mm diameter, 0.3 mm height) were prepared from inks having the same composition as those used in section 2.5 plus 10T1/2 cells at 5.0 × 10^5^ cells/mL. The effects of SFNF addition during printing were investigated by measuring the viability of cells enclosed in the printed constructs 1 h after printing and by measuring the growth profiles of the cells seeded in cell culture dishes and cultured in the DME medium. Viability was measured by staining cells using calcein-AM and propidium iodide followed by counting of the stained cells using a fluorescence microscope (BZ-9000, Keyence, Osaka Japan). The cells seeded in cell culture dishes were obtained by soaking the printed constructs in the medium containing 1 mg/mL hyaluronidase for 2 h for degrading. Furthermore, the effects of SFNFs on the viability and morphology of cells in the printed constructs were measured for about 1 week. The growth of the cells enclosed in the constructs containing gelatin-Ph was estimated by an increase in the amount of a water-soluble formazan dye derived from a tetrazolium salt dissolved in the medium containing a disk-shaped construct, using a colorimetric assay kit (Cell Counting Kit-8, Dojindo, Kumamoto, Japan). Briefly, the cell-laden construct was soaked in a 0.25 mL medium containing a 1/10 vol% reagent in the assay kit. After 6 h of incubation at 37 °C, the absorbance at 450 nm attributed to the water-soluble formazan dye was measured using a spectrophotometer. Mitochondrial activity was defined by the increase in the absorbance at 450 nm per hour.

### 2.7 Statistical analysis

Comparisons between data sets were made using an unpaired *t* test. Values of *p* < 0.05 were considered to indicate significance.

## 3 Results and discussion

### 3.1 Effect of SFNFs on solutions

**Fig. 2:**
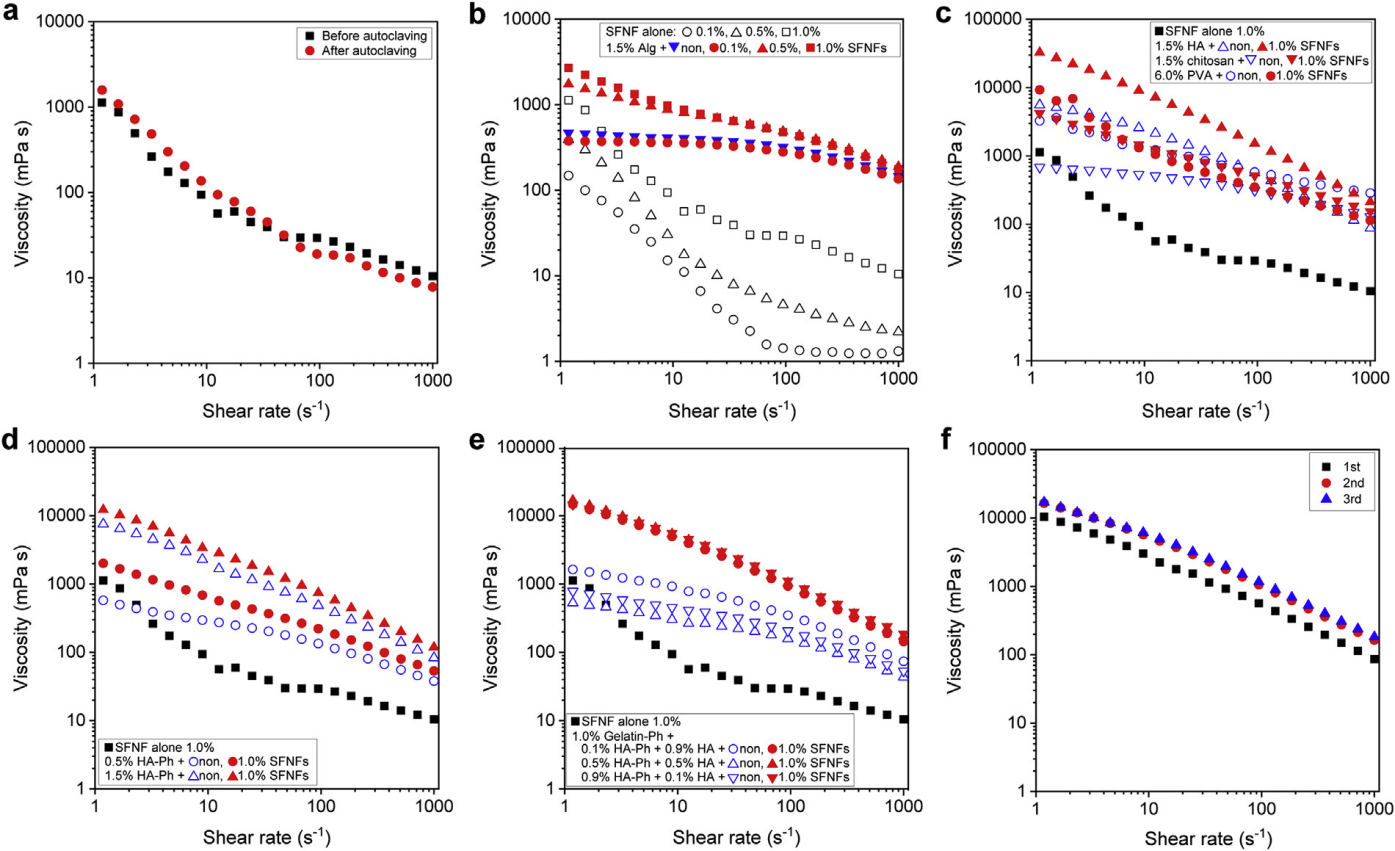
Shear rate–viscosity profiles of solutions. Effects of **a**) autoclaving PBS containing 1.0 w/v% SFNFs, **b**) SFNFs content in PBS (SFNFs alone) or in 1.5 w/v% Alg solution, **c**) anionic (1.5 w/v% HA), cationic (1.5 w/v% chitosan), and nonionic (6.0 w/v% PVA) polymers in solutions in the absence or presence of 1.0 w/v% SFNFs, **d**) HA-Ph content in solutions in the absence or presence of 1.0 w/v% SFNFs, **e**) contents of HA-Ph in the solution containing 1.0 w/v% gelatin-Ph in the absence or presence of 1.0 w/v% SFNFs, and **f**) repeated loading of shear stress to 1.0 w/v% SFNFs solution with 0.1% HA-Ph + 0.9% HA + 1.0% gelatin-Ph. HA, hyaluronic acid; Ph, phenolic hydroxyl; PBS, hosphate-buffered saline; poly(vinyl alcohol); SFNFs, silk fibroin nanofibers.

Before undertaking studies of SFNFs as an additive in bioinks, we investigated the possibility of autoclaving. The viscosities of PBS (pH 7.4) containing 1.0 w/v% SFNFs before and after autoclaving (121 °C, 20 min) were almost the same (Fig. 2a). This result demonstrates the possibility of sterilization of SFNFs by autoclaving. This feature is useful for biomedical applications including bioprinting, because treatment by autoclaving is the most common method of sterilization.

Next, we investigated the effect of the content of SFNFs on the viscoelastic properties of solutions. Alg solution containing 0.1 w/v% SFNFs showed almost the same profile as the solution free of SFNFs (Fig. 2b). However, the effect of SFNF addition became remarkable at >0.5 w/v% SFNFs. The viscosity of 1.5 w/v% Alg solution containing 1.0 w/v% SFNFs was about twice that of solution containing 0.5 w/v% SFNFs at a 1 s^−1^ shear rate. The viscosity decreased with the increasing shear rate, and the value at the shear rate 1000 s^−1^ was of a similar magnitude to that of 1.5 w/v% Alg solution free of SFNFs. This result demonstrates that the shear thinning of the Alg solution was enhanced by SFNFs at >0.5 w/v%.

Then, we studied the versatility of SFNFs for enhancement of shear thinning of solutions containing polymers with different electric charges: HA (anionic), chitosan (cationic), and PVA (neutral). As shown in Fig. 2c, the addition of SFNFs enhanced the shear thinning of these solutions independent of the electric charge of the polymer. It was also confirmed that the addition of SFNFs enhanced the shear thinning of polymer solutions regardless of the presence of Ph moieties for 0.5 w/v% and 1.5 w/v% HA-Ph solutions (Fig. 2d). Furthermore, SFNFs were effective in increasing shear thinning even in solutions containing multiple polymers. The viscosities of solutions containing 1.0 w/v% gelatin-Ph, HA-Ph, and HA at different concentrations (HA + HA-Ph = 1.0 w/v%) and 1.0 w/v% SFNFs were almost the same (Fig. 2e). The values were about 10 times larger than those for solutions free of SFNFs independent of the contents of HA-Ph and HA. As we expected, these results demonstrate the versatility of SFNFs obtained through mechanical disintegration of degummed silk fibers as an additive for enhancement of shear thinning of polymer solutions.

Regarding the mechanism of the enhancement of shear thinning by SFNFs, it may be suggested that the destruction of SFNFs by shear stress caused the decrease of viscosity at a high shear rate. However, this appears not to be the case when the solution containing SFNFs, gelatin-Ph, HA-Ph, and HA at 1.0 w/v%, 1.0 w/v%, 0.1 w/v%, and 0.9 w/v%, respectively, was subjected to loading of shear stress three times; the viscosity of the solution did not decrease but increased slightly (Fig. 2f). The mechanism of the enhancement of the shear thinning by SFNFs is considered to be the same as that for other nanofibers, including cellulose nanofibers. It is explained by the shear-induced alignment of the nanofibers [[36], [37], [38]]. Tanaka et al. [38] reported that cellulose nanofibers with a higher aspect ratio (= fiber length/fiber diameter) induced larger viscosity because longer fibers begin to align with the shear flow at a low shear rate. To date, the mechanical grinding conditions of degummed silkworm silk fibers resulting in the SFNFs with a different shape from those used in this study has not been established. The influence of the shape of SFNFs will be investigated in the future after the establishment of the manufacturing conditions. The increase of viscosity detected in the repeated loading of shear stress to the solution containing SFNFs (Fig. 2f) could be due to the change in the orientation of individual SFNFs by the shear stress from the spin of a parallel plate sandwiching the solution.

### 3.2 Effect of SFNFs on gelation time and stiffness of hydrogels

**Fig. 3:**
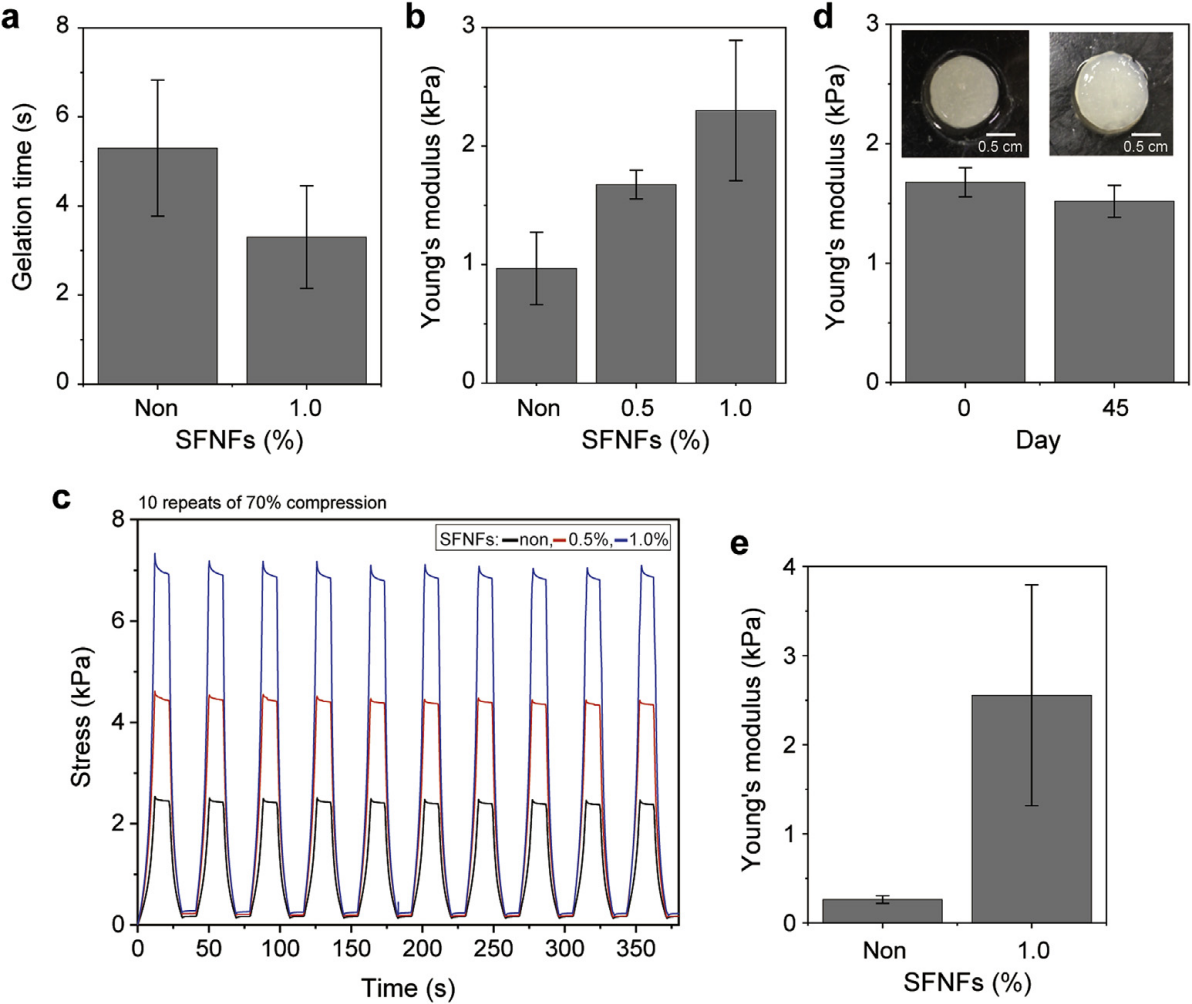
Effects of SFNFs addition on **a**) gelation and **b–e**) hydrogel mechanical properties. **a**) Gelation time of 1.5 w/v% HA-Ph solutions. **b**) The Young's modulus and **c**) repeated compression–stress profiles of hydrogels obtained from 1.5 w/v% HA-Ph solutions. **d**) Photographs and the Young's modulus of hydrogels obtained from 1.5 w/v% HA-Ph + 1.0 w/v% SFNF solution at 0 and 45 days of soaking in PBS. **e**) The Young's modulus of hydrogels obtained from 0.1 w/v% HA-Ph + 0.9 w/v% HA + 1.0 w/v% gelatin-Ph. All the solutions contained HRP at 5 U/mL. Bars in graphs: mean ± sS.D. (*n* = 4). HA, hyaluronic acid; HRP, horseradish peroxidase; Ph, phenolic hydroxyl; SFNFs, silk fibroin nanofibers; S.D., standard deviation.

It has been reported that the addition of cellulose nanofibers induced not only the enhancement of shear thinning of solutions but also an increase of the compressive modulus of resultant hydrogels [[14], [15], [16]]. Based on previous reports, we studied the effect of addition of SFNFs on the mechanical properties of hydrogels. Before the investigation, we studied the effect of SFNFs on gelation time because it has been reported that hydrogels obtained with different rates of gelation catalyzed by HRP showed different mechanical properties [[39], [40], [41]]. Fig. 3a shows the gelation times of 1.5 w/v% HA-Ph + 5 U/mL HRP solutions free of SFNFs and those containing 1.0 w/v% SFNFs. The gelation time of the solution containing SFNFs was 3.0 ± 0.3 s, and the time was similar to that for the solution free of SFNFs (*p* = 0.12). Fig. 3b shows Young's moduli of the hydrogels obtained from 1.5 w/v% HA-Ph solutions free of SFNFs and those containing 0.5 w/v% and 1.0 w/v% SFNFs. The Young's modulus increased with the increasing SFNF content, and the value for the hydrogels obtained from the solution with 1.0 w/v% SFNFs was about 2.5 times that for the solution free of SFNFs (*p* = 0.01). There was no visible change in the compression–stress profiles of the hydrogels in 10 repetitions of 70% compression, independent of SFNF content (Fig. 3c). Furthermore, a hydrogel containing 1.0 w/v% SFNFs was stable during 45 days of soaking in PBS. No obvious swelling or decrease of the Young's modulus was observed (Fig. 3d). These results demonstrate that the incorporation of SFNFs did not reduce the durability of the hydrogels. The effect of SFNFs on the mechanical properties of hydrogels was not specific to hydrogels containing HA-Ph as the sole polymeric component. Enhancement of the Young's modulus was observed for hydrogels containing multiple polymers, i.e. 1.0 w/v% gelatin-Ph, 0.1 w/v% HA-Ph, and 0.9 w/v% HA (Fig. 3e). In addition, the effect was not specific to hydrogels cross-linked by HRP-catalyzed reaction. Gelatin hydrogels obtained by cooling 7.0 w/v% gelatin solution containing 1.0 w/v% SFNFs showed a higher Young's modulus than those free of SFNFs (Fig. S1).

The effect of SFNF addition in increasing the Young's modulus of hydrogels could be explained in the same way as the addition effect of other nanofibers. Shin et al. [17] reported that gelatin methacrylamide hydrogels containing cellulose nanofibers showed a larger Young's modulus than those free of nanofibers. They explained that the nanofibers functioned as a structural frame of the hydrogels [17]. Kai et al. [42] reported a similar result for gelatin hydrogels containing electrospun poly(ϵ-caprolactone)/gelatin and explained that the strengthening of hydrogels resulted from the transfer of the load from the polymer chain to the nanofibers. The increase of the mechanical stiffness of hydrogels by adding SFNFs to solutions would not only be governed by the increase in nanofiber density. The increase of the Young's modulus by adding SFNFs at 1.0 w/v% were 1.3, 2.3, and 4.1 kPa for the hydrogels from 1.5 w/v% HA-Ph solution (Fig. 3b), 0.1 w/v% HA-Ph + 0.9 w/v% HA + 1.0 w/v% gelatin-Ph solution (Fig. 3e), and 7.0 w/v% gelatin solution (Fig. S1), respectively. The mechanism is not clear now, but a possible explanation is the difference in the degree of interaction between the polymer(s) and SFNFs.

### 3.3 Effect of SFNFs in bioprinting

**Fig. 4:**
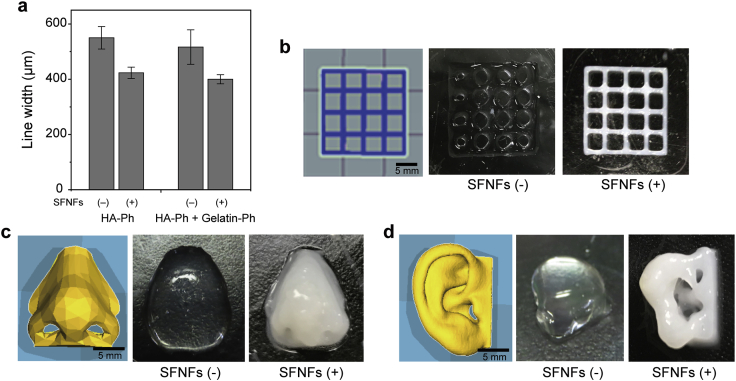
**a**) Effects of SFNF addition on the width of hydrogel filament obtained by extruding 1.5 w/v% HA-Ph (HA-Ph) and 0.1 w/v% HA-Ph + 0.9 w/v% HA + 1.0 w/v% gelatin-Ph (HA-Ph + gelatin-Ph) inks free of SFNFs (−) and containing 1.0 w/v% SFNFs (+). Bars: mean ± S.D. (*n* = 3). Blueprints and the blueprint-based hydrogels with **b**) lattice, **c**) nose, and **d**) ear shape obtained from 1.5 w/v% HA-Ph inks free of SFNFs [SFNFs (−)] and containing 1.0 w/v% SFNFs [SFNFs (+)]. The content of HRP in the inks was 5 U/mL. HA, hyaluronic acid; HRP, horseradish peroxidase; Ph, phenolic hydroxyl; SFNFs, silk fibroin nanofibers; S.D., standard deviation.

The results indicating the effectiveness of SFNF addition in enhancing shear thinning of solutions including polymers with Ph moieties suggest that it may also have a positive effect on printability. We attempted to print hydrogel constructs using an extrusion-based 3D printing system in which the inks gellable through HRP-catalyzed cross-linking were extruded onto the substrate surface in air containing H_2_O_2_ [35]. As shown in Fig. 4a, the width of the hydrogel line obtained from 1.5 w/v% HA-Ph + 5 U/mL HRP solution by extrusion onto a cell culture dish from a needle of 220 μm inner diameter and 410 μm outer diameter was about 550 μm. The width of the line decreased to 423 μm on adding SFNFs at 1.0 w/v%. The same effect of SFNF addition was found for inks composed of 0.1 w/v% HA-Ph + 0.9 w/v% HA + 1.0 w/v% gelatin-Ph. The widths of the filaments of the ink free of SFNFs and that containing SFNFs were 516 μm and 400 μm, respectively. These results show that the spreading of the extruded ink on the substrate before hydrogelation was suppressed by SFNFs, as expected from the results for the effect of SFNFs on the shear rate–viscosity profiles of solutions (Fig. 2).

The hydrogels obtained by printing the SFNFs-containing inks were more consistent with the blueprint than the hydrogels obtained from the inks without added SFNFs (Fig. 4b). Using the inks containing SFNFs, hydrogel constructs with complex configurations, such as nose- and ear-shaped hydrogels, were obtained (Fig. 4c and d). It was impossible to obtain such constructs from the inks free of SFNFs. Assessing the nose- and ear-shaped constructs obtained from ink containing SFNFs, the fidelity of the ear-shaped hydrogel to the blueprint was a little low (Fig. 4d). This is because the ear shape was more complex; it contained matrix-free space behind the helix and lobule parts and a cavity in the ear canal. To improve the fidelity of the ear-shaped hydrogel to the blueprint, optimization of conditions such as the composition of the ink, gelation rate, viscosity, extrusion rate, and the size of the needle are necessary. Finding the optimal conditions for each application is beyond the scope of this study. The important finding of the aforementioned experiments was the effectiveness of SFNFs as an additive that improved printability. Apart from the printability, a notable difference of the hydrogels free of SFNFs and containing SFNFs was their color (Fig. 4b–d). The whitish color owing to SFNFs can make microscopic imaging difficult. It could be improved by using thinner SFNFs. As described before, the manufacturing conditions for producing the SFNFs with different shapes from the SFNFs used in this study have not been established. The effect of the shape of SFNFs on the transparency of the resultant constructs will be investigated in the future after the establishment of the manufacturing conditions.

**Fig. 5:**
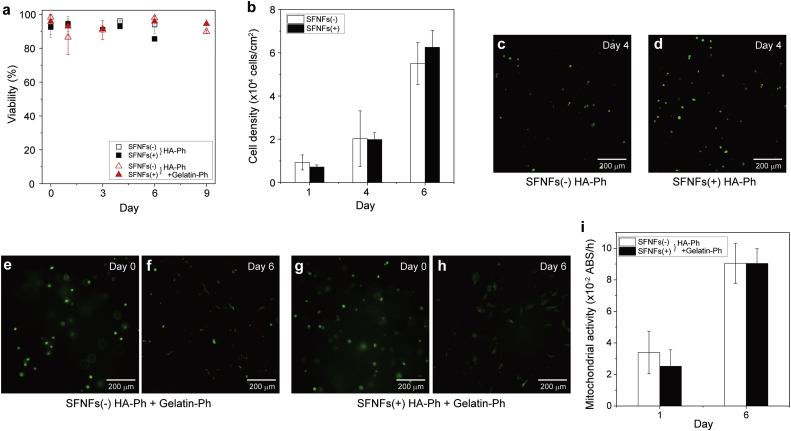
Effects of SFNF addition in inks on **a**) viability of cells enclosed in printed hydrogel constructs, **b**) growth profiles of cells collected from hydrogel constructs and then seeded on cell culture dishes, **c–h**) morphology of enclosed cells, and **i**) mitochondrial activity in cell-laden hydrogel constructs. The hydrogel constructs were obtained from 1.5 w/v% HA-Ph (HA-Ph) and 0.1 w/v% HA-Ph + 0.9 w/v% HA + 1.0 w/v% gelatin-Ph (HA-Ph + gelatin-Ph) inks free of SFNFs [SFNFs(−)] and those containing 1.0 w/v% SFNFs [SFNFs(+)]. The content of HRP in the inks was 5 U/mL. HA, hyaluronic acid; Ph, phenolic hydroxyl; SFNFs, silk fibroin nanofibers; S.D., standard deviation.

Finally, we investigated the effects of SFNFs in inks on cells by printing fibroblast 10T1/2 cell-laden constructs in the same conditions applied in the experiments shown in Fig. 4. The viabilities of the cells after 1 h of encapsulation in 1.5 w/v% HA-Ph hydrogel constructs free of SFNFs and containing SFNFs were almost the same, 92.5% and 92.7%, respectively (*p* = 0.95) (Fig. 5a). Cells were collected from the printed constructs by degradation using hyaluronidase and seeded on cell culture dishes; they showed similar growth profiles independent of the content of SFNFs (Fig. 5b). These results demonstrate the non-adverse effect of SFNFs during printing on the cells in conditions shown to be effective for improving printability (Fig. 4).

We also evaluated the effects of SFNFs on the cells enclosed in the printed constructs by measuring their viability and morphologies after longer incubation. The viabilities of the enclosed cells were >85% after 6 and 9 days of culture in HA-Ph hydrogel constructs and HA-Ph + gelatin-Ph hydrogel constructs, respectively, independent of the presence of SFNFs (Fig. 5a). The cell-laden hydrogels were printed by extruding bioinks containing cells onto the substrate surface in air containing H_2_O_2_. Therefore, it may be suggested that the heterogeneous distribution of dead cells in the hydrogels. However, such a phenomenon was not found regardless of the presence of SFNFs. This result indicates that the cytocompatibility of the bioprinting process is not reduced by SFNFs. Regarding the morphologies of 10T1/2 cells, the cells in HA-Ph hydrogels maintained a round shape independent of the presence of SFNFs (Fig. 5c and d). In contrast, the cells in the hydrogels containing gelatin-Ph had a round shape immediately after printing, but elongated in the following period independent of the presence of SFNFs (Fig. 5e–h). We assumed the growth of the cells from the elongation and measured the mitochondrial activity per hydrogel construct to estimate the growth of the cells. The values after 1 and 6 days of printing were almost the same in the presence and absence of SFNFs (Fig. 5i); the values increased about 3-fold during this period. These results demonstrate that the SFNFs did not affect cell behaviors; the cell behaviors were affected by components of the hydrogels other than SFNFs.

Regarding the elongation of cells only in the hydrogel constructs containing gelatin-Ph, it is well known that cell adhesion and elongation in/on hydrogels can be improved by incorporating gelatin or its derivatives (including gelatin-Ph) because of the presence of an amino acid sequence that mediates cell attachment (the RGD sequence) [[43], [44], [45], [46]]. HA hydrogels allow only poor adhesion of adherent cells [47,48]. Conjugation with gelatin has been reported as an effective approach for producing HA hydrogels with cell adhesion [30,[49], [50], [51]]. It is also known that surfaces composed of native silk fibroin allow poor adhesion [52,53]. Because of the attractive features of silk fibroin in tissue engineering applications, there have been various literature reports on the functionalization of silk fibroin for enhancement of cell adhesion, such as the incorporation of cell-binding peptides [54,55] and grafting of cell adhesive molecules [52]. The use of such functionalized SFNFs instead of the native SFNFs used in this study may induce the elongation of enclosed adherent cells even in HA-Ph hydrogels free of gelatin-Ph. However, the important relevant finding of this study is that the SFNFs obtained via mechanical disintegration of degummed silk fibers are inactive toward enclosed cells.

## 4 Conclusions

Silkworm silk fibroin is a promising material in the biomedical field because of its excellent biocompatibility, robust mechanical performance, ease of processing, and sufficient supply from sericulture. In the present study, we investigated the feasibility of using silk fibroin nanofibers obtained by mechanical disintegration of degummed silk fibers as an additive in bioinks for use in extrusion-based 3D bioprinting. The SFNFs functioned to enhance shear thinning of various aqueous solutions of polymers (HA, Alg, chitosan, and PVA). Furthermore, the same function was confirmed for derivatives of HA and gelatin having chemically introduced Ph moieties (HA-Ph and gelatin-Ph), which are cross-linkable through HRP-catalyzed reaction. Using inks containing the derivative(s), SFNFs, and HRP, 3D hydrogel constructs with higher fidelity to blueprints were obtained by extruding on substrates in air containing H_2_O_2_ compared with those obtained using inks free of SFNFs. The SFNFs added to enhance printability did not cause a decrease in the viability of 10T1/2 cells during printing and did not affect the behaviors of the cells. Cells enclosed in non-cell adhesive HA-Ph hydrogel constructs maintained a round shape, and cells enclosed in cell adhesive hydrogels containing gelatin-Ph elongated independent of the presence of SFNFs. From these results and the well-known attractive characteristics of silk fibroin, we conclude that SFNFs obtained by mechanical grinding of degummed silkworm silk fibers are a promising additive for bioinks as an alternative to cellulose nanofibers, which are frequently used to enhance shear thinning, to improve printability without decreasing the viability of enclosed cells.

## Data availability

All experimental data within the article and its Supplementary Information are available from the corresponding author upon reasonable request.

## Credit author statement

**S. Sakai:** Conceptualization, Methodology, Investigation, Formal analysis, Writing - original draft, Writing - review & editing, Visualization, Supervision. **A. Yoshii:** Methodology, Investigation, Validation, Writing - review & editing. **S. Sakurai:** Resources, Methodology, Writing - review & editing. **K. Horii:** Resources, Methodology, Writing - review & editing. **O. Nagasuna:** Conceptualization, Resources, Writing - review & editing.

## Declaration of competing interest

The authors declare that they have no known competing financial interests or personal relationships that could have appeared to influence the work reported in this paper.
