# Supplementary material for: Silk fibroin nanofibers: a promising ink additive for extrusion three-dimensional bioprinting
Source: Mater Today Bio. 2020 Sep 19;8:100078. doi: 10.1016/j.mtbio.2020.100078 (PMC7552084; doi:10.1016/j.mtbio.2020.100078)
Supplement: Multimedia component 1 [file mmc1.docx]

Supplementary Material

**Silk fibroin nanofibers: A promising ink additive for extrusion 3D bioprinting**

Shinji Sakai^1^*, Ayano Yoshii^1^, Shunsuke Sakurai^2^, Kazuki Horii^2^, Osamu Nagasuna^2^

^1^Division of Chemical Engineering, Department of Materials Engineering Science, Graduate School of Engineering Science, Osaka University. 1-3 Machikaneyama-Cho, Toyonaka, Osaka 560-8531, Japan

^2^Nagasuna Mayu Inc., Kyotango, Kyoto 629-3101, Japan

*E-mail: sakai@cheng.es.osaka-u.ac.jp


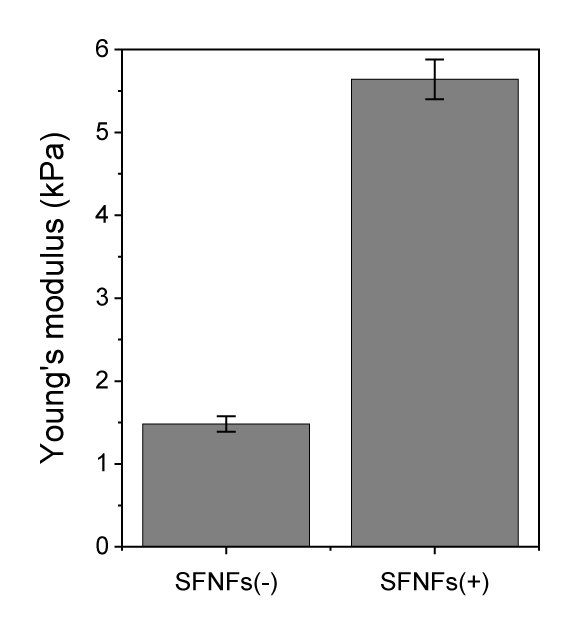


**Figure S1.** Effects of silk fibroin nanofibers (SFNFs) addition on mechanical properties of 7 w/v% gelatin hydrogels. SFNFs (−): Free of SFNFs, SFNFs (+): Containing 1 w/v% SFNFs. The hydrogels were obtained by cooling 7% gelatin aqueous solutions free of SFNFs and containing 1 w/v% SFNFs. Bars in graphs mean ± S.D. (*n* = 3).
